# A Narrative Review of Urodynamics in Men With Lower Urinary Tract Symptoms: Diagnostic Test, Treatment-Decision Tool, or Prognostic Marker?

**DOI:** 10.7759/cureus.111293

**Published:** 2026-06-22

**Authors:** Velina Banda, Deng Gang

**Affiliations:** 1 International College of Education, Zhejiang Chinese Medical University, Hangzhou, CHN; 2 Department of Urology, Hangzhou First People's Hospital, Hangzhou, CHN

**Keywords:** bladder outlet obstruction, detrusor underactivity, lower urinary tract symptoms, non-invasive assessment, urodynamics

## Abstract

Lower urinary tract symptoms (LUTS) in men are common, multifactorial, and frequently difficult to attribute to a single underlying mechanism. Although invasive urodynamic studies (UDS), particularly pressure-flow studies, have long been considered the reference standard for physiological characterization of bladder outlet obstruction (BOO), detrusor underactivity (DU), and mixed dysfunction, their routine use has come under increasing scrutiny in recent years. This narrative review evaluates the contemporary role of UDS in male LUTS as a diagnostic tool, treatment-decision aid, and prognostic marker. It also examines non-invasive alternatives, current controversies, and future directions.

Current practice increasingly supports moving away from routine preoperative UDS in men with uncomplicated LUTS toward a more selective, indication-driven approach. UDS continues to offer important value in situations with greater diagnostic uncertainty, including young men with refractory symptoms, suspected DU, persistent symptoms after previous intervention, and neurogenic lower urinary tract dysfunction, where accurate assessment of storage pressures, compliance, and sphincter coordination may be important for upper urinary tract protection.

Pressure-flow studies remain uniquely capable of differentiating BOO from impaired detrusor contractility and identifying mixed dysfunction when non-invasive tests are inconclusive. Non-invasive tools such as uroflowmetry, post-void residual measurement, ultrasound-derived parameters, and clinical prediction models may serve as useful triage instruments within a stepwise diagnostic pathway, but they have not yet replaced invasive UDS in complex or high-risk patients. Technical quality, standardization, and interobserver variability continue to represent important limitations of urodynamic testing. Contemporary practice, therefore, favors a selective approach to UDS in male LUTS, with its greatest clinical utility lying in the resolution of meaningful diagnostic uncertainty and support of individualized, phenotype-driven management rather than broad routine application.

## Introduction and background

Lower urinary tract symptoms (LUTS) are common among men and constitute a major reason for consultation in urological practice [1]. With increasing age, voiding, storage, and post-micturition symptoms become more frequent [2]. These symptoms may affect quality of life, sleep, sexual function, and general well-being [1,3]. Although benign prostatic obstruction (BPO) remains an important contributor in older men, the pathophysiology of male LUTS is often multifactorial. Symptoms may reflect bladder outlet obstruction (BOO), detrusor underactivity (DU), detrusor overactivity (DO), impaired bladder compliance, dysfunctional voiding, neurogenic dysfunction, or mixed abnormalities [4]. This heterogeneity creates a persistent diagnostic challenge because non-invasive tests, including uroflowmetry and post-void residual (PVR) volume measurement, often cannot reliably identify the dominant underlying mechanism [4,5].

Urodynamic studies (UDS), particularly pressure-flow studies and videourodynamics (VUDS), have traditionally been regarded as the reference standard for objective physiological assessment of lower urinary tract function [6]. By measuring bladder pressure and urinary flow during filling and voiding, UDS can distinguish BOO from impaired detrusor contractility, identify mixed dysfunction, characterize storage-phase abnormalities, and, when combined with imaging, localize sites of functional obstruction [7]. For many years, this detailed physiological information was expected to improve diagnostic accuracy, guide treatment selection, especially before prostate surgery, and predict postoperative outcomes [8].

However, the routine use of UDS has faced increasing scrutiny [9]. Current European Association of Urology and American Urological Association guidelines advise against systematic urodynamic testing in uncomplicated non-neurogenic male LUTS and recommend its use mainly when the diagnosis remains uncertain or when results are likely to influence management [4,10]. This position reflects growing recognition that additional physiological data does not automatically translate into improved clinical outcomes [11].

The UPSTREAM randomized controlled trial provided high-level evidence supporting selective rather than routine UDS. In men with bothersome LUTS being considered for surgery, adding invasive UDS to routine clinical assessment did not improve symptom outcomes or reduce surgical rates compared with standard evaluation alone [9,12]. These findings challenged the assumption that routine preoperative UDS improves treatment decision-making in broad male LUTS populations. They also shifted the debate away from whether UDS is universally valuable and toward the more clinically relevant question of when it adds value. This distinction reinforces the difference between physiological accuracy and clinical utility.

The contemporary role of UDS is therefore best understood as targeted rather than routine [9,11]. Evidence suggests that UDS retains important value when clinical uncertainty is high, particularly in men with suspected DU, young men with refractory LUTS, patients with neurogenic lower urinary tract dysfunction (NLUTD), those with discordant non-invasive findings, and individuals with persistent symptoms after previous intervention [4,10]. In these settings, UDS may refine diagnosis, support risk stratification, guide shared decision-making, and help establish realistic treatment expectations.

Importantly, the value of UDS is not singular. UDS may serve at least three related but distinct roles in the evaluation of male LUTS. First, it functions as a diagnostic investigation by clarifying the underlying pathophysiology when symptoms and routine non-invasive tests are insufficient. Second, it may act as a treatment-decision tool by influencing patient selection, surgical planning, and preoperative counselling. Third, it has been investigated as a prognostic marker because pretreatment urodynamic findings may help estimate symptom improvement, voiding outcomes, catheter independence, or postoperative success. Although these roles overlap conceptually, they are not equivalent, and failure to distinguish them has contributed to persistent confusion regarding the true clinical utility of UDS. These three clinical roles are summarized in the table below.

This narrative review examines the contemporary role of UDS in men with LUTS by evaluating these three related but distinct functions: its role as a diagnostic test, a treatment-decision tool, and a prognostic marker. Particular attention is given to selected patient populations, including men with DU, young men with persistent LUTS, and patients with neurogenic dysfunction. By synthesizing evidence from randomized trials, systematic reviews, and targeted cohort studies, this review aims to clarify when UDS adds value beyond standard assessment and how it can be used appropriately within modern, patient-centered urological practice.

For this narrative review, relevant literature was identified through searches of PubMed, Google Scholar, and major urological guideline documents. Search terms included combinations of "male lower urinary tract symptoms", "urodynamics", "pressure-flow study", "videourodynamics", "bladder outlet obstruction", "detrusor underactivity", "non-invasive urodynamics", "uroflowmetry", "post-void residual", and "benign prostatic obstruction". Priority was given to current guidelines, randomized controlled trials, systematic reviews, meta-analyses, and clinically relevant cohort studies. Additional references were identified from the bibliographies of selected articles. Studies were selected according to their relevance to the diagnostic, treatment-decision, or prognostic role of UDS in men with LUTS.

## Review

Urodynamics as a diagnostic tool

The most established role of UDS in male LUTS is diagnostic. LUTS are defined by symptoms, but symptoms alone do not reliably identify the underlying pathophysiological mechanism. Clinical urodynamics is designed to reproduce patient symptoms while objectively measuring lower urinary tract function during filling and voiding [7]. This allows functional abnormalities to be assessed when symptom scores, physical examination, uroflowmetry, or PVR measurement are insufficient.

The diagnostic value of UDS is greatest in selected patients with atypical, complex, or treatment-refractory LUTS. These include men with suspected DU, younger men whose symptoms are not explained by prostate size or routine non-invasive findings, and patients with NLUTD [4]. In these groups, management often depends on the accurate identification of the dominant mechanism of dysfunction. UDS can clarify whether symptoms are related to BOO, impaired detrusor contractility, storage-phase dysfunction, dysfunctional voiding, impaired compliance, or mixed abnormalities [6].

Diagnostic value in suspected DU

DU is a clear example of this diagnostic challenge. It remains clinically important but difficult to define and diagnose consistently. Its terminology, epidemiology, diagnostic criteria, and clinical boundaries remain imperfectly standardized [13]. No simple and reproducible non-invasive method can currently diagnose DU with confidence [14]. This limitation is clinically important because DU may occur alone, coexist with BOO, or be mistaken for BOO when patients present with low flow and incomplete emptying [6].

Pressure-flow studies provide the most direct physiological method for distinguishing DU from BOO [4]. Their diagnostic strength lies in the simultaneous measurement of detrusor pressure and urinary flow during voiding. Detrusor pressure reflects the force generated by the bladder muscle, while flow rate reflects the effectiveness of bladder emptying through the outlet. A low maximum flow rate may result from BOO, DU, poor voiding effort, or mixed dysfunction. In BOO, the detrusor often generates high pressure against an obstructed outlet, whereas in DU, detrusor pressure and flow are usually both reduced. This pressure-flow relationship makes pressure-flow study more informative than symptoms, uroflowmetry, or PVR volume alone [6,15-18].

Standardized indices further support diagnostic interpretation. Bladder outlet obstruction index (BOOI), calculated as PdetQmax-2×Qmax, quantifies outlet resistance, while bladder contractility index (BCI), calculated as PdetQmax+5×Qmax, estimates detrusor strength [19]. When used together, these indices help stratify patients into clinically relevant categories, including unobstructed patients with impaired contractility and patients with mixed BOO and DU. The pressure-flow plot also provides a visual representation of pressure and flow patterns throughout voiding, helping place numerical values within a broader physiological context [17].

In men with suspected DU, this objective assessment is particularly valuable because subjective symptom severity correlates poorly with actual detrusor contractility [11]. Without pressure-flow studies, patients may be misclassified, leading to inaccurate diagnosis and unrealistic treatment expectations. A man with low flow and high residual urine may be presumed to have BOO when impaired contractility is the dominant problem [6]. Conversely, some obstructed patients maintain a reasonable flow rate by generating high detrusor pressure, making obstruction less apparent on uroflowmetry alone. Pressure-flow studies therefore help confirm impaired detrusor contractility, estimate its severity, and clarify whether it exists alone or together with BOO [20]. This distinction is important for diagnosis, counselling, and treatment planning, particularly because men with impaired contractility may respond less predictably to outlet-relieving procedures [21].

Diagnostic value in young men with LUTS

Young men with LUTS represent a distinct diagnostic group compared with older men. In men under 50 years, the etiology is more heterogeneous and includes primary bladder neck obstruction (PBNO), dysfunctional voiding, DU, and DO, while BPO is less common [22]. Symptoms, uroflowmetry, and symptom scores often fail to accurately predict the underlying mechanism. As a result, many young men receive prolonged empirical treatment for chronic prostatitis, overactive bladder, or presumed early prostatic obstruction without a confirmed diagnosis [22].

Urodynamic evaluation, particularly VUDS, offers high diagnostic value in this population. A systematic review reported a urodynamic diagnosis in 79% of young men using conventional UDS compared with 98% using VUDS [23]. Targeted studies in selected patients with persistent symptoms or low uroflow have shown even higher diagnostic yields [22]. The most common diagnoses in young men differ from those in older patients. PBNO is frequently identified, with reported rates of 41-47% across major series, followed by dysfunctional voiding [22,24,25]. DU is less prevalent but remains relevant, while BPO is relatively uncommon, especially in men under 40 years.

A key advantage of VUDS over a conventional pressure-flow study is its ability to localize the site of obstruction. Conventional UDS may overdiagnose DU when anatomical localization is unavailable, whereas VUDS more commonly identifies PBNO in young men [22,23]. Anatomical visualization can therefore reclassify cases initially labelled as impaired contractility. Consistent with findings in older men, symptom patterns correlate poorly with urodynamic diagnoses in young patients. Irritative or obstructive symptoms are unreliable predictors of DO or BOO, respectively [26].

Urodynamic testing frequently changes management in this group. Multiple studies report diagnostic and therapeutic shifts in 46-80% of selected young men, enabling mechanism-directed treatment such as alpha-blockers or bladder neck incision for PBNO, pelvic floor biofeedback for dysfunctional voiding, and avoidance of unnecessary outlet surgery in DU [27]. Despite the high diagnostic yield in appropriately selected patients, routine UDS or VUDS is not recommended for all young men with LUTS. The strongest indication exists in those with persistent or treatment-refractory symptoms, abnormal uroflowmetry, or high voiding symptom scores or when surgical intervention is being considered. Non-invasive parameters, particularly abnormal uroflow, can help enrich the population likely to benefit [24].

Urodynamic assessment, particularly VUDS, provides important diagnostic value in carefully selected young men with LUTS. Its greatest strength lies in identifying the underlying mechanism and localizing functional obstruction, thereby guiding targeted therapy and reducing inappropriate treatment. A selective approach offers the best balance between diagnostic benefit and avoidance of unnecessary invasive testing.

Diagnostic value in NLUTD

NLUTD represents one of the clearest and strongest indications for UDS in men. Unlike uncomplicated non-neurogenic LUTS, where the primary goal is symptom relief, management in NLUTD focuses on protecting the upper urinary tract, preventing urinary tract infections, avoiding unsafe storage pressures, and preserving quality of life [4,10]. These objectives cannot be reliably achieved through symptoms or non-invasive tests alone because neurogenic bladder dysfunction is frequently complex, unpredictable, and poorly correlated with neurological level or clinical presentation [28].

The central limitation in NLUTD is the weak correlation between neurological findings, patient-reported symptoms, and actual lower urinary tract physiology. Clinical neurological examination alone often fails to predict urodynamic behavior. VUDS, by contrast, provides functional and anatomical information that is essential for individualized management [29].

The greatest value of UDS in this population lies in the detection of unfavorable urodynamic parameters associated with upper urinary tract deterioration. These include neurogenic DO, detrusor-sphincter dyssynergia (DSD), poor bladder compliance, elevated storage pressures, high detrusor leak point pressure, and vesicoureteral reflux. Storage detrusor pressures ≥40 cm H₂O and compliance <20 mL/cm H₂O are widely accepted high-risk thresholds. In a prospective study of 97 patients with acute spinal cord injury, 90% demonstrated at least one unfavorable parameter within the first year, with DO plus DSD present in 88% and storage pressures ≥40 cm H₂O in 39% [30]. These data underscore why symptom-guided management alone is considered unsafe.

Spinal cord injury best illustrates the need for early and serial urodynamic assessment. Unfavorable findings can appear much earlier than traditionally expected, with up to two-thirds of patients showing high-risk features within 40 days after injury [31]. Bladder behavior also evolves over time, making longitudinal monitoring clinically important. Although classic lesion-level patterns exist, such as suprasacral lesions typically causing DO with DSD and sacral lesions causing acontractile bladder, significant individual variation occurs. Management therefore cannot rely on the neurological level alone [32].

The utility of UDS extends beyond spinal cord injury. Distinct patterns are seen in multiple sclerosis, Parkinson's disease, and multiple system atrophy, where UDS can aid both management and differential diagnosis [33]. UDS is especially critical in patients unable to report symptoms reliably, such as those in a minimally conscious state. In one cohort, every patient showed unfavorable findings on VUDS, resulting in major treatment changes in more than half of the patients [34]. Beyond initial diagnosis, UDS plays a vital role in risk stratification, treatment selection, and long-term monitoring. Systematic reviews confirm that reduced compliance and high detrusor leak point pressure are major risk factors for upper tract damage, particularly in spinal cord injury and spina bifida [35]. Urodynamic findings directly influence decisions regarding clean intermittent catheterization, pharmacological therapy, botulinum toxin injections, and major reconstructive surgery.

Major guidelines endorse UDS in neurogenic patients, particularly when upper tract risk is a concern [4,10]. However, limitations remain. The test is invasive and resource-intensive, and the strongest evidence comes from spinal cord injury cohorts. Evidence quality is more limited in other neurological conditions, and optimal follow-up intervals are not firmly established.

UDS occupies a central and evidence-based role in the management of NLUTD. By identifying high-risk bladder behavior that cannot be predicted by symptoms or neurological examination, UDS enables proactive management aimed at preserving renal function and improving long-term outcomes. VUDS is particularly advantageous in NLUTD because it combines pressure measurements with fluoroscopic imaging, allowing the simultaneous assessment of bladder pressure, sphincter coordination, reflux, and anatomical abnormalities [7]. The main selected patient groups in whom UDS provides diagnostic value beyond routine non-invasive assessment are summarized in the table below.

Non-invasive alternatives as triage tools rather than replacements for urodynamics

Growing interest in non-invasive alternatives reflects the limitations of invasive UDS, including patient discomfort, infection risk, cost, procedural complexity, and the artificial nature of laboratory-based testing [4,17]. Although pressure-flow studies remain the reference standard for physiological characterization of lower urinary tract dysfunction, non-invasive tools may improve patient selection and reduce unnecessary invasive investigations [36]. Their current value lies mainly in triage within an integrated diagnostic pathway rather than in replacing UDS.

Uroflowmetry remains the most widely used first-line non-invasive assessment in men with LUTS because it is simple, inexpensive, and easy to perform [37]. It provides useful screening information about voiding function, particularly Qmax, voided volume, and flow-curve pattern. However, its diagnostic specificity is limited. An abnormal flow pattern can indicate impaired voiding, but it cannot reliably identify the underlying cause [17].

Interpretation of uroflowmetry is influenced by several physiological, technical, and patient-related factors. Flow measurements may vary according to bladder volume, detrusor contractility, outlet resistance, abdominal straining, patient position, anxiety, and testing conditions [7]. Both DU and BOO can produce reduced or flattened flow curves, limiting the ability of uroflowmetry to distinguish impaired contractility from increased outlet resistance. Flow-curve morphology is also vulnerable to artifacts and subjective interpretation, contributing to interobserver and intraobserver variability [38].

More recent quantitative approaches to flow-curve analysis may improve diagnostic discrimination. Irregular, fluctuating, or interrupted voiding patterns appear more common in impaired detrusor contractility and dysfunctional voiding than in pure BOO. Additional flow-derived parameters may provide more detailed information about voiding dynamics [39]. Nevertheless, these approaches remain supportive rather than definitive. Uroflowmetry can identify abnormal voiding and guide the need for further evaluation, but combined pressure-flow measurement remains necessary when precise differentiation between DU and BOO is required [40].

Non-invasive pressure-flow testing has been evaluated as a catheter-free method for assessing voiding function in men with LUTS/benign prostatic hyperplasia (BPH). The UroCuff Test estimates isovolumetric bladder pressure (Pcuff) via penile cuff interruption during natural voiding, with results interpreted on the Newcastle Noninvasive Nomogram. In a large retrospective multicenter study of 50,680 men, Kaplan et al. demonstrated significant age-related changes in Pcuff, Qmax, voided volume, flow rate efficiency, and PVR. Pcuff increased with age, peaking around 62 years before declining, consistent with progressive bladder compensation followed by decompensation. While these findings support the utility of non-invasive pressure-flow testing for evaluating bladder performance and outlet obstruction, the study's cross-sectional, clinic-based design and lack of symptom or outcome data limit causal inferences and generalizability. Non-invasive testing remains a promising adjunctive tool but does not replace conventional invasive urodynamics when detailed physiological characterization is needed [41].

Ultrasound-derived parameters provide another practical adjunct to clinical evaluation. Measurements such as bladder wall thickness, detrusor wall thickness, ultrasound-estimated bladder weight, and intravesical prostatic protrusion may help stratify patients according to the likelihood of BOO or bladder dysfunction [42,43]. These parameters are clinically relevant because bladder wall hypertrophy can reflect detrusor thickening and increased bladder workload, particularly in conditions associated with chronic outlet resistance, DO, or neurogenic dysfunction.

Ultrasound parameters may also have value in monitoring treatment response and estimating risk [42]. Bladder wall hypertrophy may decrease after relief of obstruction, whereas persistent hypertrophy after treatment has been associated with ongoing symptoms and poorer outcomes. In symptomatic men with BPO, bladder wall hypertrophy has also been linked to a higher risk of acute urinary retention [43]. These observations support the use of ultrasound as a complementary tool in evaluating bladder remodeling and potential disease progression.

However, ultrasound-derived measurements remain adjunctive rather than definitive. Their interpretation is limited by methodological variability, including differences in probe position, bladder filling volume, measurement site, and the specific parameter used. These differences have produced varying reference values and diagnostic thresholds, limiting direct comparison across studies. Bladder filling volume is particularly important because detrusor wall thickness changes during filling and may decrease rapidly at lower bladder volumes before reaching a more stable plateau [42].

These limitations become especially relevant in advanced or decompensated bladder dysfunction. In men with chronic urinary retention or hypocontractile detrusor function, structural bladder changes may not reliably predict postoperative recovery. Even conventional investigations may struggle to determine whether bladder emptying will improve after outlet surgery when long-standing obstruction has led to complex functional impairment [44]. Therefore, although ultrasound-derived parameters can improve risk stratification and support clinical decision-making, they cannot replace pressure-flow studies when precise differentiation among BOO, DU, mixed dysfunction, impaired compliance, and progressive bladder failure is required [40].

PVR measurement is another commonly used adjunct in men with LUTS. Elevated residual urine may indicate inefficient bladder emptying and can help identify patients who require closer assessment [4]. However, PVR is not disease-specific. It cannot independently distinguish BOO from impaired detrusor contractility, poor bladder sensation, or mixed dysfunction. For this reason, PVR is best interpreted as supportive information rather than as a definitive diagnostic measure.

Symptom questionnaires and multivariable clinical prediction models may also improve initial triage. By combining symptoms, uroflowmetry, prostate size, PVR, and other clinical variables, some models achieve moderate discriminatory performance in differentiating BOO from DU in selected cohorts [45-47]. Nevertheless, symptom severity generally shows only weak to moderate correlation with urodynamic findings. Even well-performing prediction tools, therefore, remain insufficient for definitive physiological characterization, especially when invasive or irreversible treatment is being considered [17].

Ambulatory urodynamic monitoring has been explored as another approach to improving physiological assessment. Traditional ambulatory monitoring may offer advantages over conventional laboratory UDS because it allows bladder filling under more natural conditions [48]. However, it still usually requires catheterization and remains limited by patient burden, technical complexity, and availability. Newer remote and wireless approaches are being developed to allow longer-term, catheter-free monitoring of bladder pressure and volume during daily activities. These technologies may eventually provide more representative and patient-friendly data on lower urinary tract function, but they remain investigational in many settings. Their clinical value will depend on validation in diverse patient populations, standardization of measurement methods, and evidence that they improve diagnosis or management beyond existing pathways [49-51].

Non-invasive assessments are therefore best understood as complementary triage tools within a stepwise diagnostic pathway. They may help identify low-risk patients, support preliminary risk stratification, and clarify which patients require invasive UDS. The practical goal is not to replace invasive UDS entirely, but to use non-invasive tools more effectively before deciding whether invasive testing is necessary. This approach may reduce unnecessary testing while preserving diagnostic accuracy in patients with complex, discordant, or high-risk presentations. Common non-invasive tools and their role in relation to UDS are summarized in the table below.

UDS as a treatment-decision tool in men with LUTS

UDS has long been used in men with LUTS/BPO with the expectation that objective physiological assessment can improve treatment selection. The rationale is clinically logical: if UDS can distinguish BOO from DU, identify mixed dysfunction, and assess storage-phase abnormalities, it should help clinicians choose appropriate treatment and avoid ineffective intervention. This distinction is particularly relevant before prostate surgery because outlet-relieving procedures are most likely to benefit patients whose symptoms are mainly driven by obstruction, whereas men with predominant DU or complex bladder dysfunction may have less predictable outcomes [52,53].

The strongest evidence challenging routine UDS as a treatment-decision tool comes from the UPSTREAM trial. This large pragmatic multicenter randomized controlled trial enrolled 820 men with bothersome LUTS in whom surgery was being considered and compared routine clinical assessment with routine assessment plus invasive UDS. At 18 months, patient-reported outcomes were similar between groups, and surgery rates were almost identical, with 38% in the UDS arm and 36% in the routine-care arm undergoing surgery. The UDS pathway was non-inferior for symptom outcomes, but it did not reduce surgery rates or show a clear advantage in postoperative symptom improvement [9]. These findings indicate that routine preoperative UDS does not provide measurable benefit for all men with uncomplicated LUTS being considered for surgery [11].

However, UPSTREAM should not be interpreted as evidence that UDS has no role in treatment decision-making. Rather, it shows that its value is selective. In men with straightforward clinical findings, standard assessment may already provide enough information to guide treatment. In such cases, additional physiological testing may not change management in a clinically meaningful way. Exploratory analyses from UPSTREAM suggest that UDS may be more useful in selected subgroups, particularly when non-invasive findings are less clear. Urodynamic measures appeared to have greater predictive value in men with relatively preserved urinary flow, especially when Qmax was above 15 mL/s, where BOO or impaired contractility may be less obvious clinically [12]. These findings support a targeted approach in which UDS is reserved for patients whose treatment pathway remains uncertain after standard evaluation.

Evidence from systematic reviews supports this interpretation. A Cochrane review found that invasive urodynamic testing changed clinical management more often than clinical assessment alone, with management changes occurring in 13% of men in the UDS group compared with none in the control group [11]. However, the quality of evidence was low, and there was no clear evidence that these management changes led to better symptom relief, improved quality of life, or superior objective outcomes such as increased flow rate. This creates an important clinical paradox: UDS can change decisions, but a change in decision does not automatically translate into better patient outcomes. Therefore, the usefulness of UDS should be judged not only by whether it alters management but by whether that change is likely to benefit the individual patient.

One of the most important treatment-decision roles of UDS is differentiating BOO from DU. Clinical variables such as symptom scores and PVR are not sufficient to distinguish these conditions reliably, yet the distinction can strongly influence surgical counselling and expectations [53]. Meta-analytic evidence suggests that preoperative DU is associated with poorer improvement after transurethral surgery, including smaller improvements in International Prostate Symptom Score (IPSS) and Qmax [54]. This supports the use of UDS when impaired contractility is suspected and when the expected benefit of outlet-relieving surgery is uncertain. At the same time, DU should not be regarded as an absolute contraindication to surgery. Some men with DU still benefit from outlet procedures, especially when BOO coexists and when residual detrusor function or bladder sensation is preserved [20].

This nuance is central to the treatment-decision role of UDS. Its purpose is not simply to determine whether surgery should be performed. In many cases, its greater value lies in refining risk assessment and guiding shared decision-making. Men with clear BOO and preserved contractility can be counselled about a higher likelihood of symptomatic and objective improvement. In contrast, men with DU, absent sensation, or mixed dysfunction can be counselled more cautiously regarding incomplete symptom relief, persistent PVR, or possible catheter dependence. This makes UDS especially useful when the clinical question is not only whether surgery is possible but what outcome is realistic for the individual patient [52,53].

UDS also has value in specific clinical scenarios where routine assessment is often insufficient. In young men with refractory LUTS, the underlying diagnoses differ from those commonly seen in older men. PBNO, dysfunctional voiding, impaired detrusor contractility, and BPO may all occur, but they cannot be reliably separated by symptoms alone. VUDS is particularly useful in this setting because it combines pressure-flow studies with fluoroscopic visualization, allowing more accurate localization of obstruction and better differentiation between functional outlet disorders and impaired contractility. Without urodynamic evaluation, young men may be treated empirically for chronic prostatitis or overactive bladder, often without clear benefit [22-27].

In men with neurogenic LUTS or complex neurological conditions, UDS may be even more important for treatment planning. Patients with spinal cord disorders, Parkinson's disease, stroke, or other neurological conditions may have BOO, DU, DO, poor compliance, DSD, or mixed dysfunction. These findings carry different implications for surgery, catheterization, pharmacological treatment, botulinum toxin injections, or other interventions. UDS or VUDS may also be necessary in frail elderly patients with urinary retention or overflow incontinence, where the main clinical question is whether symptoms reflect obstruction, impaired detrusor function, or both [34]. Similarly, in men with persistent or worsening symptoms after previous prostate surgery, UDS can help determine whether symptoms are caused by residual obstruction, poor detrusor contractility, DO, or another mechanism [33-35].

UDS may also support patient-centered decision-making. Some patients prefer to have as much diagnostic information as possible before choosing surgery, especially when the expected benefit is uncertain or when the risks of intervention are meaningful [9]. In this context, UDS can improve counselling by providing a clearer explanation of the underlying dysfunction. This is particularly relevant in patients with mixed storage and voiding symptoms, where the dominant mechanism may not be obvious from symptoms alone. A patient with storage-predominant symptoms may still have significant BOO, while a patient with voiding-predominant symptoms may have DU, DO, dysfunctional voiding, or bladder hypersensitivity [55]. UDS can therefore help align treatment choice with the actual physiological abnormality rather than with symptom labels alone.

Despite these advantages, several limitations restrict the routine use of UDS. The test is invasive, uncomfortable for some patients, and associated with cost, time, and the need for trained staff and dedicated equipment. Reported complications include bacteriuria, urinary tract infection, gross hematuria, acute urinary retention, pain during the procedure, and transient irritative symptoms afterward [56]. These burdens are important because routine testing is difficult to justify when the expected clinical benefit is small. Current guidelines therefore do not recommend UDS as a routine evaluation for all men with LUTS and increasingly limit its use to cases where the diagnosis remains uncertain after standard assessment [4,10].

The central controversy remains the gap between improved physiological information and improved clinical outcomes. UDS may discourage surgery in some patients, identify DU in others, or confirm BOO in selected cases, but available evidence does not consistently show that these changes improve outcomes at the population level [9]. This does not mean that UDS is unhelpful. It means that its value depends heavily on patient selection. The best candidates are those in whom the result is likely to change treatment choice, surgical planning, risk counselling, or patient expectations. Examples include men with borderline uroflowmetry, mixed storage and voiding symptoms, or suspected DU or young men with refractory LUTS, neurogenic LUTS, failed prior surgery, or strong patient preference for maximum diagnostic clarification before intervention.

Current evidence supports UDS as a selective treatment-decision tool rather than a routine preoperative requirement. In men with clear clinical features of obstruction and concordant non-invasive findings, routine UDS is unlikely to add enough value to justify its burden. In contrast, in patients with equivocal findings, complex presentations, suspected impaired contractility, or previous treatment failure, UDS can provide information that directly informs treatment planning and counselling. Until stronger non-invasive alternatives are validated, the most evidence-based approach is not to abandon UDS, but to use it carefully when its findings are likely to meaningfully influence management [9,31].

UDS as a prognostic marker

The most ambitious proposed role of UDS is as a prognostic tool capable of predicting treatment response and postoperative outcomes in men with LUTS. This role is particularly attractive before invasive therapies such as prostate surgery, where more accurate prediction of benefit could improve patient selection, refine counselling, and reduce unnecessary or poorly targeted interventions [57]. Despite this strong physiological rationale, the prognostic performance of individual urodynamic parameters has been variable and often inconsistent across studies.

A central limitation is that lower urinary tract dysfunction in men with LUTS is rarely driven by a single isolated mechanism. Symptom improvement after treatment depends on the interaction of several factors, including the degree of BOO, detrusor contractility, compensatory bladder function, bladder compliance, storage-phase abnormalities, bladder sensation, age, comorbidities, and duration of obstruction [56]. As a result, single urodynamic parameters frequently show only modest predictive accuracy when used in isolation [17,18]. Uroflowmetry parameters such as Qmax correlate imperfectly with symptom severity and surgical outcomes because of overlap between obstructed and non-obstructed patients. Similarly, pressure-flow study parameters such as PdetQmax, BOOI, and BCI have shown inconsistent predictive value across different populations [6,58,59].

Nevertheless, certain urodynamic patterns carry clinically meaningful prognostic implications. Men with clear BOO and preserved detrusor contractility generally experience the most favorable outcomes after deobstructive surgery, including greater improvements in IPSS, Qmax, PVR, and quality of life. In contrast, patients with significant DU, poor bladder compliance, absent or reduced bladder sensation, or mixed dysfunction tend to have more variable and less predictable responses [53].

DU illustrates these prognostic challenges particularly well. Historically, DU was often considered a relative contraindication to outlet surgery because of concerns regarding poor postoperative voiding recovery and persistent retention [60,61]. Contemporary evidence, however, presents a more nuanced picture [40]. Although DU is associated with poorer outcomes on average, including smaller improvements in IPSS and Qmax, many men with DU still achieve meaningful symptomatic and functional benefit after outlet-relieving procedures. Benefit appears more likely when some degree of contractility and bladder sensation is preserved and when BOO coexists [20]. Conversely, severe contractile impairment combined with poor compliance, absent sensation, and very high residual urine volumes is associated with higher rates of incomplete emptying and long-term catheter dependence [54].

Even within DU populations, traditional urodynamic parameters often have limited ability to predict individual outcomes. Studies evaluating postoperative catheter-free voiding have found that measures such as PdetQmax, BCI, and PVR do not consistently predict surgical success [62,63]. This reflects a broader limitation of current prognostic approaches: lower urinary tract dysfunction exists along a continuum, yet many studies rely on rigid threshold-based categorization, such as BCI <100 for DU [17]. Patients with apparently similar urodynamic profiles may therefore experience markedly different postoperative trajectories.

Storage-phase abnormalities provide additional prognostic information. Reduced cystometric capacity, impaired bladder compliance, and DO are associated with an increased risk of persistent storage symptoms, including urgency, frequency, and nocturia, after outlet surgery [57]. However, these relationships are not absolute. Some men with preoperative DO improve substantially after outlet relief, suggesting that obstruction itself may contribute to storage dysfunction in a subset of patients [16].

The prognostic utility of UDS is particularly relevant in complex and high-risk populations. In men with NLUTD, poor bladder compliance and elevated storage pressures are strong predictors of upper urinary tract deterioration and long-term renal risk. These parameters often predict risk more reliably than symptoms or neurological level alone [30,35]. In non-neurogenic but complex cases, the combination of DO and impaired contractility may identify a difficult mixed dysfunction phenotype associated with poorer voiding outcomes and persistent LUTS. In men with persistent symptoms after previous prostate surgery, UDS can help differentiate residual obstruction from de novo or unmasked DU, thereby guiding further management decisions [64,65].

Age and comorbidities further modify prognostic interpretation. Although detrusor contractility tends to decline with advancing age, chronological age alone is a poor independent predictor of surgical response. Older men with clear BOO and reasonable contractility may still achieve satisfactory outcomes, whereas younger men with severe DU or dysfunctional voiding may experience disappointing results despite technically successful surgery [66].

An important conceptual distinction therefore emerges. UDS rarely functions as a precise standalone predictor capable of generating reliable individual prognostic scores. Its greater clinical value lies in risk stratification and expectation management rather than deterministic outcome prediction. By identifying unfavorable physiological patterns, UDS can support more individualized counselling, help set realistic expectations, and improve shared decision-making before irreversible interventions.

The prognostic role of UDS in male LUTS is selective and context-dependent. Integrated assessment of obstruction, contractility, compliance, sensation, and storage function can provide useful insight into probable treatment response and potential risk. However, current evidence does not support reliance on isolated urodynamic parameters as definitive predictors of surgical success. Used as part of a broader multifactorial evaluation, UDS remains useful for refining risk assessment and supporting patient-centered decision-making.

Table 1 presents the three related but distinct clinical roles of UDS in the evaluation of male LUTS.

**Table 1:** Clinical roles of UDS in men with LUTS BOO: bladder outlet obstruction; DO: detrusor overactivity; DU: detrusor underactivity; LUTS: lower urinary tract symptoms; NLUTD: neurogenic lower urinary tract dysfunction; UDS: urodynamic studies Table Credits: Velina Banda. This table was created by the author to summarize the major clinical roles of UDS in contemporary male LUTS evaluation. The reference citations included within each row indicate the sources supporting the corresponding role, contribution, target population, and limitation.

Role of UDS	Main clinical question	Key urodynamic contribution	Best-use population	Main limitation
Diagnostic test	What is the underlying mechanism of LUTS?	Differentiates BOO, DU, DO, impaired compliance, dysfunctional voiding, and mixed dysfunction [6,7,17,18]	Young men with suspected DU, refractory LUTS, NLUTD, and discordant non-invasive findings [13,14,22,23,28,29]	Invasive, technically dependent, and not needed routinely in uncomplicated cases [4,9,11]
Treatment-decision tool	Will UDS change management or counselling?	Helps guide surgery, conservative management, catheterization, pharmacotherapy, or patient counselling [9,11,12,52,53]	Equivocal cases, suspected impaired contractility, prior failed surgery, and young men with functional obstruction [20,22,23,52,53]	Management changes do not always translate into improved clinical outcomes [9,11]
Prognostic marker	What outcome is realistic after treatment?	Identifies risk patterns such as DU, poor compliance, absent sensation, DO, and high storage pressures [35,54,57,61-63]	Men considering outlet surgery, DU patients, men with NLUTD, and patients with persistent LUTS after surgery [30,35,54,57,64,65]	Isolated urodynamic parameters have limited individual predictive accuracy [17,18,57]

Table 2 summarizes the main selected patient groups in whom UDS provides diagnostic value beyond routine non-invasive assessment.

**Table 2:** Patient groups in which UDS has selective diagnostic value BOO: bladder outlet obstruction; BPO: benign prostatic obstruction; DO: detrusor overactivity; DSD: detrusor-sphincter dyssynergia; DU: detrusor underactivity; LUTS: lower urinary tract symptoms; NLUTD: neurogenic lower urinary tract dysfunction; PBNO: primary bladder neck obstruction; PVR: post-void residual; Qmax: maximum urinary flow rate; UDS: urodynamic studies; VUDS: videourodynamics Table Credits: Velina Banda. This table was created by the author to summarize selected clinical populations in whom UDS or VUDS may provide diagnostic information beyond symptoms and routine non-invasive assessment. The reference citations included within each row indicate the sources supporting the corresponding patient group, diagnostic limitation, urodynamic value, and clinical impact.

Patient group	Why routine assessment may be insufficient	Main UDS/VUDS value	Clinical impact
Men with suspected DU	Symptoms, Qmax, and PVR cannot reliably distinguish DU from BOO [13,15,16]	Confirms impaired detrusor contractility and identifies coexisting BOO [6,17,18,20]	Improves counselling and surgical expectation setting, particularly before outlet-relieving procedures [20,53,59]
Young men with refractory LUTS	BPO is less common; PBNO, dysfunctional voiding, DU, and DO are more likely [22-27]	VUDS localizes functional obstruction and distinguishes PBNO from dysfunctional voiding or DU [22-25]	Enables mechanism-directed treatment and avoids prolonged empirical therapy [22,23,27]
Men with NLUTD	Symptoms and neurological level may not predict bladder physiology [28,29]	Detects DO, DSD, poor compliance, high storage pressures, and reflux [29-35]	Supports renal protection and long-term risk management [30,35]
Men with persistent symptoms after prostate surgery	Symptoms may reflect residual obstruction, DU, DO, or mixed dysfunction [64,65]	Identifies whether symptoms are due to persistent obstruction or bladder dysfunction [64,65]	Guides reoperation decisions or non-surgical management [64,65]
Men with discordant non-invasive findings	Symptoms, prostate size, Qmax, and PVR may not align [4-6,36,46,47]	Provides objective pressure-flow clarification [6,17,18]	Helps decide whether invasive treatment is appropriate [4,9,11,12]

Table 3 provides a summary of the common non-invasive tools and their role in relation to UDS.

**Table 3:** Non-invasive alternatives and their role in urodynamic triage AI: artificial intelligence; BOO: bladder outlet obstruction; BWT: bladder wall thickness; DU: detrusor underactivity; DWT: detrusor wall thickness; IPP: intravesical prostatic protrusion; IPSS: International Prostate Symptom Score; LUTS: lower urinary tract symptoms; PVR: post-void residual; Qmax: maximum urinary flow rate; UDS: urodynamic studies; UEBW: ultrasound-estimated bladder weight Table Credits: Velina Banda. This table was created by the author to summarize commonly used and emerging non-invasive tools in the evaluation of men with LUTS. The reference citations included within each row indicate the sources supporting the corresponding tool, function, strength, limitation, and role in relation to UDS.

Non-invasive tool	What it provides	Strengths	Main limitation	Role in relation to UDS
Uroflowmetry	Qmax, voided volume, and flow-curve pattern [37-39]	Simple, inexpensive, and widely available [37,38]	Cannot reliably distinguish BOO from DU and is affected by voided volume, effort, and artifacts [37-39]	First-line screening and triage tool [36-39]
PVR measurement	Residual urine after voiding [4,5,36]	Easy to perform and useful as a marker of emptying efficiency [4,5]	Non-specific and cannot identify the underlying mechanism [4,5,36]	Supportive risk marker rather than a mechanistic diagnostic test [4,5,36]
Symptom scores/IPSS	Symptom burden and bother [3,37,45]	Standardized and patient-centered [3]	Weak correlation with urodynamic diagnosis [37,45]	Baseline assessment tool, not a substitute for mechanistic diagnosis [37,46]
Ultrasound-derived parameters	Prostate size, IPP, BWT, DWT, and UEBW [42-45]	Adds anatomical and morphological information [42-45]	Thresholds and measurement methods vary [42]	Risk stratification and triage tool [36,42-45]
Prediction models/AI tools	Combined clinical and non-invasive prediction [40,47,58]	May improve selection for UDS [40,47,58]	Require external validation and prospective testing [40,47,58]	Emerging adjunct for patient selection [40,47,58]
Ambulatory/catheter-free monitoring	Physiological bladder pressure or volume data during daily activity [48-51]	Potentially more patient-friendly and realistic than conventional laboratory testing [48-51]	Still investigational in many settings [48-51]	Future adjunct, not routine replacement for invasive UDS [48-51]

Current concepts and controversies

The role of UDS in male LUTS has shifted from routine preoperative testing toward selective, indication-driven use [4,9,17]. This change reflects an important distinction between physiological accuracy and clinical utility. UDS can characterize lower urinary tract dysfunction in detail, but this does not mean that routine testing improves outcomes in all patients. The central contemporary debate is therefore not whether UDS can measure bladder and outlet function, but when this information meaningfully changes management, counselling, or prognosis [9,11,12].

The UPSTREAM trial and related analyses have strongly influenced this shift. In men with uncomplicated LUTS being considered for surgery, adding invasive UDS to standard assessment did not improve symptom outcomes, reduce surgical rates, or improve quality of life [9]. These findings challenged the assumption that routine preoperative physiological assessment would improve treatment selection in broad LUTS populations. However, they should not be interpreted as an argument against UDS in all settings. The main implication is that UDS should be used when the result is likely to answer a specific clinical question rather than as a routine step before intervention.

This selective approach creates a practical controversy because the threshold for an "appropriate" indication is not always clear. Some clinicians favor broader physiological assessment before irreversible treatment, especially when there is concern for DU, mixed dysfunction, or discordant non-invasive findings. Others argue that the limited outcome benefit shown in randomized trials does not justify invasive testing outside clearly selected cases. This difference in interpretation contributes to variation across institutions and healthcare systems regarding when UDS is performed.

Technical quality is another major concern. The value of UDS depends on accurate pressure measurement, correct calibration, appropriate test conditions, reliable signal interpretation, and minimization of artifacts [7,17,18]. Pressure drift, incorrect zeroing, signal artifacts, and interpretation variability can lead to the inaccurate classification of BOO, DU, or mixed dysfunction. These limitations are particularly important in borderline cases, where small technical errors may change diagnostic classification. Updated International Continence Society (ICS) standards provide a framework for improving performance and reporting, but consistent implementation remains challenging outside specialized centers [17,18].

The rise of non-invasive alternatives and computational tools has added another layer to the debate. Uroflowmetry, PVR, ultrasound-derived parameters, non-invasive pressure-flow testing, clinical prediction models, and machine learning approaches may improve triage and help identify patients who are more likely to require invasive assessment [36]. Among these, non-invasive pressure-flow testing, including penile cuff-based methods such as the UroCuff Test, is particularly relevant because it attempts to approximate pressure-flow information without catheter-based measurement [41]. However, these approaches remain adjunctive rather than definitive, particularly in complex or high-risk patients where precise physiological differentiation is required.

Patient-centered care is also increasingly relevant. UDS may cause discomfort, anxiety, embarrassment, or concern about infection, and these burdens must be weighed against expected clinical benefit. At the same time, some patients value the additional diagnostic explanation provided by physiological testing, particularly when symptoms are persistent or unexplained or when surgery is being considered [25]. Shared decision-making therefore requires a clear discussion of what question the UDS is expected to answer, how the result may change management, and what limitations the test has.

The move toward selective testing also does not apply equally across all patient groups. In NLUTD and in some frail or high-risk patients, the purpose of UDS extends beyond symptom explanation. The goal may include identifying unsafe storage pressures, poor compliance, DSD, reflux, or other findings that threaten upper urinary tract function. In these populations, UDS remains more central because clinically important abnormalities may be missed by symptoms and routine non-invasive tests alone [28-35].

Current controversies, therefore, center less on whether UDS is physiologically valid and more on how it should be integrated into modern care. The most important unresolved questions concern patient selection, technical standardization, cost-effectiveness, patient acceptability, and the role of non-invasive triage tools. Future progress will depend on combining selective invasive testing with reliable non-invasive assessment, standardized quality control, and phenotype-driven interpretation of male LUTS.

Future directions

Future research should move beyond the broad question of whether UDS is useful for all men with LUTS. The more important task is to identify the specific clinical settings in which invasive physiological assessment provides meaningful benefit [9,11,12]. Refinement of patient selection remains a major priority, particularly through the integration of clinical variables, uroflowmetry, PVR, imaging findings, and computational approaches that can help identify patients most likely to benefit from invasive testing [36,40,47].

Improved technical standardization is also essential. Multicenter studies continue to show variability in UDS performance and interpretation, highlighting the need for wider implementation of updated ICS quality standards, standardized reporting systems, and ongoing operator training [17,18]. Greater attention to calibration, artifact recognition, pressure signal quality, and interpretation consistency may improve diagnostic reliability and strengthen confidence in clinical decision-making [17,18].

Artificial intelligence and machine learning approaches represent a rapidly developing area. Early studies using advanced uroflowmetry analysis, flow-curve morphology, ultrasound-derived parameters, and integrated prediction models have shown promise for identifying BOO, DU, and abnormal voiding patterns non-invasively [36,39,40,47]. However, external validation across diverse populations and healthcare systems remains limited [40,47]. Future studies should therefore prioritize prospective validation and pragmatic evaluation of whether these tools improve patient selection, reduce unnecessary invasive testing, and maintain diagnostic safety.

Greater attention should also be given to the phenotype-based classification of male LUTS. Future models will likely need to move beyond isolated urodynamic thresholds and symptom-based categories [17,20,57]. More useful approaches may integrate obstruction, contractility, compliance, sensation, storage-phase abnormalities, age, comorbidities, prostate anatomy, and patient-reported outcomes. This may allow more nuanced counselling and more individualized treatment pathways than current symptom-based frameworks alone.

The future role of UDS will likely depend on refinement rather than expansion. Its value will be strongest when used within a stepwise diagnostic pathway that combines non-invasive triage, technically reliable invasive testing, and individualized interpretation. Such an approach may preserve the diagnostic strengths of UDS while reducing unnecessary testing in patients unlikely to benefit.

## Conclusions

UDS should be understood as a selective clinical tool rather than a routine investigation for all men with LUTS. Its greatest value lies in clarifying the underlying mechanism of symptoms when routine assessment is insufficient, particularly in patients with diagnostic uncertainty, suspected impaired detrusor function, complex presentations, persistent symptoms after treatment, or neurogenic dysfunction.

The key role of UDS is not simply to provide more physiological information, but to support better diagnosis, counselling, treatment selection, and risk assessment in carefully selected patients. Non-invasive assessments remain useful for initial evaluation and triage, but UDS continues to have an important place when precise physiological clarification is needed to guide individualized, phenotype-driven management.
